# VOILA on the LUVMI-X Rover: Laser-Induced Breakdown Spectroscopy for the Detection of Volatiles at the Lunar South Pole

**DOI:** 10.3390/s22239518

**Published:** 2022-12-06

**Authors:** David S. Vogt, Susanne Schröder, Lutz Richter, Michael Deiml, Peter Weßels, Jörg Neumann, Heinz-Wilhelm Hübers

**Affiliations:** 1 Deutsches Zentrum für Luft- und Raumfahrt e.V. (DLR), Institut für Optische Sensorsysteme, 12489 Berlin, Germany; 2 OHB System AG, 82234 Weßling, Germany; 3 Large Space Structures GmbH, 85386 Eching, Germany; 4 Laser Zentrum Hannover e.V. (LZH), 30419 Hannover, Germany; 5 Institut für Physik, Humboldt-Universität zu Berlin, 12489 Berlin, Germany

**Keywords:** LIBS, laser-induced breakdown spectroscopy, spectroscopy, elemental analysis, in situ analysis, planetary science, Moon, lunar south pole, solar system exploration, regolith

## Abstract

The project Lunar Volatiles Mobile Instrumentation—Extended (LUVMI-X) developed an initial system design as well as payload and mobility breadboards for a small, lightweight rover dedicated for in situ exploration of the lunar south pole. One of the proposed payloads is the Volatiles Identification by Laser Analysis instrument (VOILA), which uses laser-induced breakdown spectroscopy (LIBS) to analyze the elemental composition of the lunar surface with an emphasis on sampling regolith and the detection of hydrogen for the inference of the presence of water. It is designed to analyze targets in front of the rover at variable focus between 300 mm and 500 mm. The spectrometer covers the wavelength range from 350 nm to 790 nm, which includes the hydrogen line at 656.3 nm as well as spectral lines of most major rock-forming elements. We report here the scientific input that fed into the concept and design of the VOILA instrument configuration for the LUVMI-X rover. Moreover, we present the measurements performed with the breadboard laboratory setup for VOILA at DLR Berlin that focused on verifying the performance of the designed LIBS instrument in particular for the detection and quantification of hydrogen and other major rock forming elements in the context of in situ lunar surface analysis.

## 1. Introduction

In the dawn of several missions to the lunar surface that are planned by different nations in the coming years, resource scouting for in situ resource utilization (ISRU) is a topic of high interest for the Moon [1]. The lunar south pole is of particular interest for upcoming lunar exploration endeavors due to the detection of large reservoirs of water ice in the pole’s permanently shadowed regions by orbiting spacecrafts (e.g., [2,3]). Water from the Moon is an important resource both for life support and for potential usage as fuels and propellants for spacecrafts and could be utilized to reduce the costs of a sustained presence on the lunar surface [4]. Additionally, from a scientific point of view, the in situ detection of water is of high relevance: open questions remain about the formation and history of the lunar water, also giving insight into the formation and evolution of the Moon [5,6]. A strong focus of future robotic exploration missions will, therefore, be on the detection of water and related volatiles. 

In the framework of the Lunar Volatiles Mobile Instrumentation—Extended (LUVMI-X) project, a mid-size, low mass rover with subsystems was developed, dedicated to scout and study volatiles in lunar polar regions [7,8,9]. An integrated ion trap mass spectrometer with a drill and a neutron spectrometer allow for the analysis of volatiles in the shallow subsurface and up to a meter in depth [10]. For scouting volatiles in the surficial lunar regolith but also to analyze the composition of the regolith itself, a laser-induced breakdown spectroscopy (LIBS) instrument called Volatiles Identification by Laser Analysis (VOILA) is part of the LUVMI-X payload. LIBS permits rapid in situ multi-elemental analysis requiring optical access only and is particularly sensitive to all kinds of metal [11]. Additionally, light elements such as hydrogen can be detected. A laser is used to ablate a small amount of the sample of interest and turns it into a luminous micro plasma which is then analyzed spectroscopically. The laser-induced plasma depends highly on ambient conditions such as the pressure [12], but suitable LIBS data can be obtained in vacuum conditions such as on the Moon [13,14]. A LIBS instrument can serve as a primary scientific tool for independent sample analysis but also as a reconnaissance tool to quickly identify potentially interesting targets for further analysis with more laborious and time-consuming contact instruments or for guiding the selection of samples to be returned to Earth. The three currently active rovers on Mars (ChemCam on NASA’s Curiosity, SuperCam on NASA’s Perseverance, and MarSCoDe on the CNSA’s Zhurong) all feature LIBS instruments for the analysis of the Martian surface [15,16,17,18,19,20,21]. The first LIBS instrument developed for the Moon was onboard the Indian Pragyan rover in the framework of the Chandrayaan-2 mission [22] that, however, failed to achieve a soft landing in September 2019. The Martian LIBS instruments are all comparable in mass (about 10 kg) and capability since SuperCam is a direct enhanced upgrade of the first LIBS instrument ChemCam by the same team and the MarSCoDe design was heavily inspired by ChemCam, too. These instruments feature a telescope and a focusing system enabling analysis of Martian rocks and soils up to 7 m from the spacecraft. In contrast, the Indian LIBS for the Moon was much less performant but also much more lightweight since it was designed to analyze the lunar regolith in only 20 cm distance vertically below the rover without a focusing mechanism.

In order to allow for scouting and for sampling multiple positions in front of the LUVMI-X rover, the VOILA LIBS instrument was developed with a performance and versatility degree between the very capable but heavy Martian LIBS instruments and the very lightweight but low-performant Chandrayaan-2 LIBS instrument. The project partners from industry, OHB System AG and Laser Zentrum Hannover e.V. (LZH), and the German Aerospace Center’s (DLR) Institute of Optical Sensor Systems (OS) shared the tasks of its development, distributed into the development of the optical head (OHB), the development of a low temperature LIBS laser (LZH), and the implementation of the VOILA laboratory breadboard model and testing its performance (DLR-OS).

In this paper, we report the scientific aspects that fed into design decisions of the VOILA instrument for in situ regolith analysis on the lunar surface with a focus on hydrogen detection and quantification. Moreover, results obtained with the laboratory breadboard setup of VOILA analyzing Moon-relevant samples in experimentally simulated low pressure are presented. The results are discussed in the context of further improving the design and performance of the instrument and for its potential use as a volatile-scouting instrument at the lunar south pole.

## 2. Design Considerations

The major design choices for the VOILA LIBS instrument were A. the positioning of the LIBS instrument on the robotic platform together with the working distance, B. the degrees of freedom for moving and pointing the LIBS instrument, and C. the wavelength range and resolution of the spectrometer. A. and B. translate into the need of a specific integrated focusing system and considerably affect the mass and volume of the overall system. While for the decisions on A. and B. mostly engineering arguments were of relevance, C. was approached highly scientifically driven. Moreover, visual close-up context information of the target and the sampled position is typically needed for improved scientific interpretation of the LIBS data, translating into D. the need of an integrated camera or another camera of the rover covering the sampled position with illumination of the potentially shadowed surface. Thoughts and considerations for the points A., B., and C. are summarized in the following subsections.

### 2.1. Accommodation and Working Distance (A. and B.)

Most versatility can be obtained with a LIBS instrument mounted on the mast of a rover, as can be seen from the active ChemCam and SuperCam instruments on NASA’s Curiosity and Perseverance rovers [15,16,18,19]. They allow for measurements in distances of up to several meters from the respective rovers with telescopic systems, however, weighing about 10 kg on rovers with masses of about 1 t. The first LIBS instrument to be employed on the Moon on board the Pragyan rover of India’s Chandrayaan-2 mission [22] was much more lightweight with only about 1 kg but also much less performant: with no focusing system, this instrument would have measured the lunar regolith in a constant distance of about 20 cm below the rover’s bottom chassis. Deviations from the ideal focusing distance typically result in reduced LIBS data quality.

Sampling loose regolith is more challenging for LIBS since LIBS works best and is best interpretable when applied on solid surfaces. The laser couples less efficiently on loose soil, resulting in lower quality spectra. Sufficient irradiance on the sample is required to assure the creation of a LIBS plasma in vacuum translating into requirements for the laser and focusing optics. VOILA got specifically designed and tested to provide good quality spectra of loose regolith in vacuum.

The LIBS instrument for the LUVMI-X instrument suite was conceived to lie between the quite capable but large and massive Mars LIBS instruments ChemCam and SuperCam and the modest Indian LIBS for the Pragyan rover of Chandrayaan-2. It is based on pre-developments carried out by OHB (then Kayser-Threde), LZH, and DLR for a robotic-arm carried LIBS once considered for the ESA ExoMars rover, which was later abandoned.

### 2.2. Wavelength Range (C.)

The capability for the detection of hydrogen as an indicator of water or ice was the main objective for the VOILA LIBS instrument and therefore constituted the central scientific requirement. Although oxygen is present in most mineral phases and is not unique to sampling water, the capability of detecting oxygen with the VOILA instrument was favored, too, since an intense oxygen detection together with the detection of hydrogen would enhance the reliability of the finding. With LIBS, hydrogen and oxygen can be detected in the near infra-red (NIR) wavelength region: H(I) at 656 nm and O(I) at 777 nm and 844 nm. H(I) at 656 nm is known as hydrogen alpha line and is typically the best observable hydrogen emission in the conventional wavelength range for LIBS spanning from the ultra-violet (UV) to the NIR [23,24]. The hydrogen beta line at 486 nm is much less intense.

Another promising approach for the detection of H and O on bodies without an absorbing atmosphere is by analyzing the wavelength range in the vacuum-UV (VUV), where also other volatile elements such as C, N, S, P, and Cl have strong emission lines [25]. Due to their high excited electronic energy levels, these elements do not have strong emission lines in other wavelength ranges, and it is very challenging to detect them even in close to ideal conditions for LIBS such as on Mars. At very low pressures such as on the Moon, they were not observed within the typically applied wavelength range 220–800 nm [14]. In the VUV, hydrogen could be detected at 121.6 nm from the strong Lyman-alpha emission line. However, even though the VUV wavelength range is promising for volatile element detection for bodies without an atmosphere such as the Moon [25], for VOILA we choose the more conventional wavelength range covering the 656 nm H(I) emission as a less risky development closer and therefore with more heritage from other LIBS instruments that were already developed and flown. 

Another aspect was that in the VUV wavelength range, many major rock forming elements cannot be detected. In order to increase the value and potential scientific return of a scouting LIBS instrument, it should not only serve volatile detection but also be capable to more generally analyze the composition of the regolith and rocks on the lunar surface. The six elements that comprise the majority of oxides in lunar material are Si, Al, Ti, Fe, Mg, and Ca. These elements are all detectable with LIBS in Moon-like vacuum. Additionally, alkali elements can be detected, allowing for instance to identify KREEP-like compositions with the K emission line at 767 and 770 nm among others [14]. However, the different major elements of the lunar surface appear over a wide wavelength range in the LIBS data from the UV to the NIR. All LIBS instruments on Mars use three different spectrometers combined to cover the wavelength range from the UV to the NIR and allow for suitable resolution of the spectral lines. For LUVMI-X, a compromise had to be made for good coverage of the most important emission lines but only with one spectrometer in order to reduce mass for a not too heavy instrument for the mid-size LUVMI-X mobile platform. A decision had to be made regarding which elements are of highest priority to be detected in accordance with the major scientific objectives of LUVMI-X. For instance, LIBS can also be optimized for the detection of potentially interesting mineral phases for extracting consumables such as O_2_ and H_2_O. Ilmenite (FeTiO_3_) is one of the promising candidates for oxygen production on the Moon. Titanium can be best observed from the UV to about 500 nm. Iron emission is best observed in a similar range but more shifted towards the UV. On the other hand, engineering aspects were weighted in, such as aiming for relatively easily available optical components and coatings and therefore not extending the wavelength range too far towards the UV.

## 3. VOILA Design

**Figure 1:**
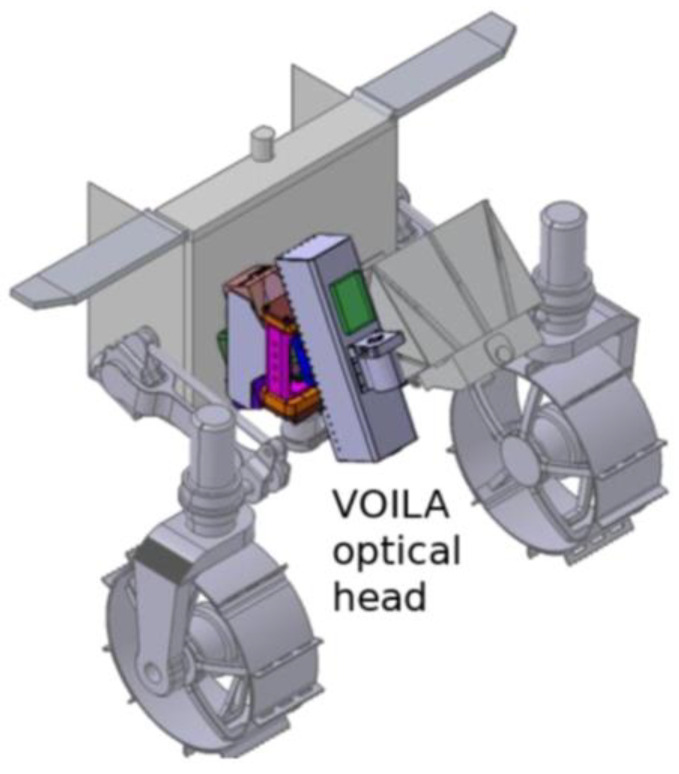
CAD model of the accommodation of the VOILA optical head at the front of the LUVMI-X rover. It can be tilted to the left and right, enabling the sampling of several positions to the front of the rover.

Figure 1 shows a CAD model of the optical head of VOILA mounted at the front of the LUVMI-X rover’s body. The instrument is divided into two parts: the movable optical head and a fixed unit comprising the spectrometer and electronics. The optical head includes the LIBS laser and a focusing mechanism to achieve working distances from 300 mm to 500 mm. It can be rotated along one axis in order to aim at different targets on the lunar surface in front of the rover. Focusing is achieved with an integrated LED illuminating the target of interest and a camera (the forward-facing LUVMI-X hazard-detection cam) detecting the spot on the surface and deriving the distance. The light collected from the laser-induced plasma is coupled into a fiber that guides it into the spectrometer in the rover body. The spectrometer then records the spectrum in the wavelength range from 350 nm to 790 nm, which includes the H emission lines at 486.1 nm and 656.3 nm as well as emission lines of the major rock-forming elements (Si, Ti, Al, Fe, Mg, Ca, Na, K, and O).

## 4. VOILA Breadboard Model at DLR-OS

**Figure 2:**
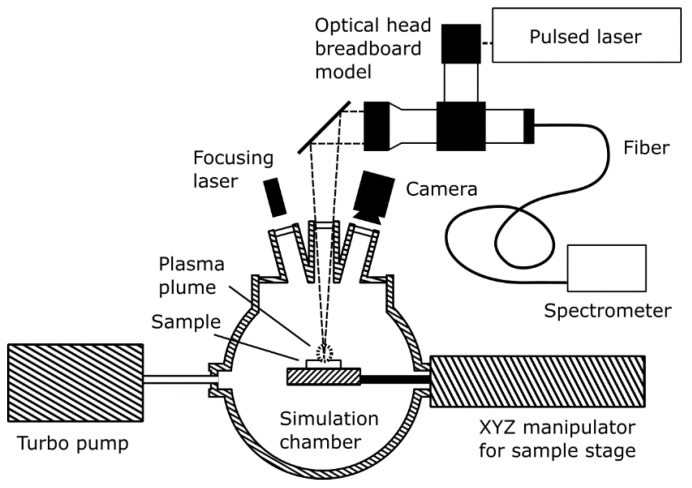
Diagram of the VOILA laboratory demonstration model. The breadboard optical head was built by OHB from COTS components. The laser was developed by LZH in the framework of the LUVMI-X project. Integration of the components and combination with coolable vacuum chamber was performed by and at DLR-OS.

**Figure 3:**
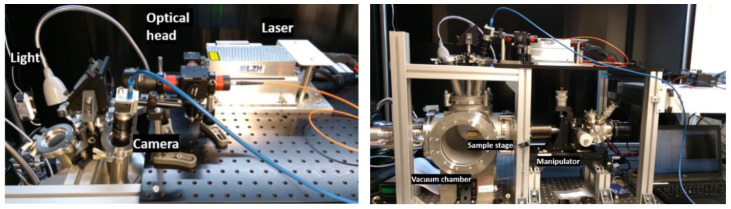
Pictures of the VOILA laboratory demonstration model. (**Left**): laser and optical head can be seen, which guide the laser pulses onto the sample in the vacuum chamber (**right**).

The VOILA laboratory setup at DLR-OS consists of the VOILA breadboard model and the vacuum chamber. A sketch of the setup is shown in Figure 2; an image of the setup can be seen in Figure 3. The vacuum chamber uses a turbo pump to reach pressures lower than 1 mPa, which is sufficiently close to lunar ambient conditions to enable representative LIBS measurements. Inside the chamber, the sample is placed onto a sample stage, which can be moved with the XYZ manipulator to adjust the position of the sample. An inspection camera provides a live feed of the inside of the chamber and a small laser module can be used to find the focus position of the sample.

**Figure 4:**
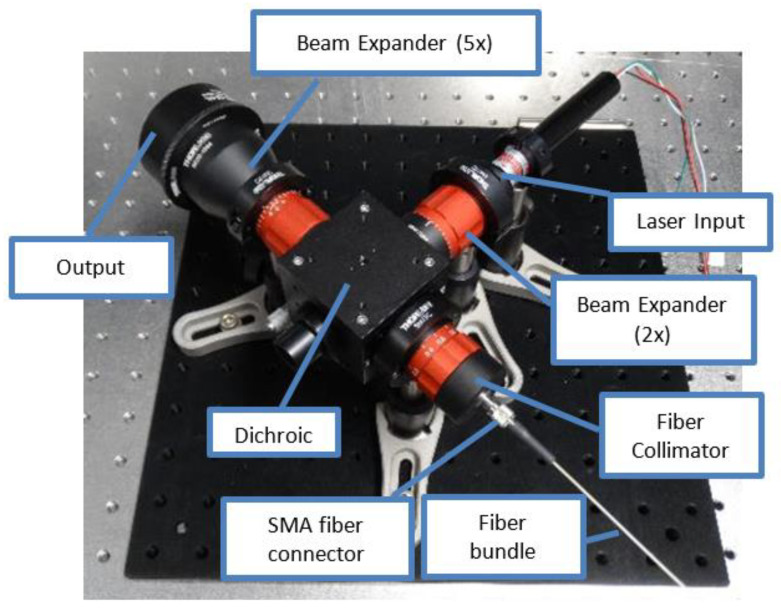
Optical head assembled from COTS components.

The VOILA breadboard model is mounted on top of the vacuum chamber. It consists of the pulsed Yb:YAG laser developed by the Laser Zentrum Hannover e.V., an optical head assembled from commercial off-the-shelf components (see Figure 4), and a fiber-coupled compact spectrometer (Avantes AvaSpec-Mini). The Yb:YAG laser has a wavelength of 1030 nm, a pulse duration of 7.8 ns, and a pulse frequency of 10 Hz. The pulse energy can be adjusted from 0 mJ to approximately 25 mJ. The laser beam is guided into the optical head, where it is expanded to a diameter of approximately 50 mm. The beam is then focused onto the sample inside the simulation chamber at a working distance of 400 mm, where it ignites a laser-induced plasma plume. In vacuum conditions, the plasma plume is conic with a bright region close to the ablation spot and disappears within a few nanoseconds.

The light emitted by the plasma is collected by the same optics that are used for the laser beam, but a dichroic mirror is used to split off the plasma light and guide it into the multimode fiber bundle that connects to the spectrometer. The second arm of the bifurcated fiber bundle leads to an LED that can be used to illuminate the sample. The spectrometer has a wavelength range from 340 nm to 900 nm and a spectral resolution of about 0.4 nm. However, the effective wavelength range is limited to approximately 350 nm to 800 nm due to the transmission of the optical head.

## 5. Experiments

Different studies were performed using the VOILA laboratory setup at DLR-OS in order to investigate the capabilities of VOILA. For all measurements, a low pressure of less than 10 mPa was used in order to simulate the low pressure on the lunar surface. The pulse energy of the laser was set to 17 mJ/pulse.

The first set of measurements was focused on lunar analogue samples, which include lunar regolith simulants as well as relevant geological samples provided by the Museum für Naturkunde Berlin (MfN). The main goal of these measurements was to investigate the capability of VOILA to detect the major elements in these samples and to identify minerals based on their spectra measured by VOILA. As lunar regolith simulants, we used the commercially available simulants LMS-1 and LHS-1 by Exolith, which we pressed into pellets for the measurements shown here. The natural samples provided by the MfN are granular ilmenite as well as rock fragments of dunite, anorthosite, oligoclase, and of a rock with both goethite and limonite features. The granular ilmenite was pressed into a pellet for the measurements shown here. A summary of the Exolith samples along with the expected major elements to be detected is given in Table 1. For the regolith simulants LMS-1 and LHS-1, the listed elements are those where the associated oxide has an abundance larger than 1 wt% in the sample and K_2_O, which has a strong LIBS signal and can therefore be observed even at lower concentrations. All used samples are summarized in Table 2. The exact compositions of the natural samples provided by the MfN are not known, so that the listed major elements are those that are expected for these rocks and minerals.

The second set of experiments focuses on the detection and quantification of water in lunar ambient conditions via the hydrogen line at 656.3 nm in the LIBS spectrum. This is especially challenging, because the water content in the sample is affected by adsorption of atmospheric water in the laboratory and by the evaporation of liquid water at the reduced pressures used for these experiments. Liquid nitrogen can be used to freeze samples in order to avoid the evaporation of water, but in this case the accumulation of water ice on the sample surface during the sample preparation process will be even more severe. We therefore decided to use frozen samples of water ice mixed with LMS-1 for qualitative investigations of the hydrogen signal at large water concentrations, where the effect of atmospheric water is negligible. For low water concentrations, we used gypsum (CaSO_4_·2H_2_O) as a proxy for water. The bound water in the gypsum does not evaporate at low pressures, while unwanted additional water does evaporate. Furthermore, we mixed the gypsum with sodium sulfate (Na_2_SO_4_), which provides a simple sample matrix that is very similar to the gypsum while also not being affected by water adsorption. In order to obtain a calibration at low water concentrations, mixtures of gypsum and sodium sulfate were prepared with gypsum ranging from 0 wt% to 38.4 wt%, so that the water content in the samples increased steadily from 0 wt% to 8 wt%.

## 6. Results

### 6.1. Lunar Analogue Measurements

**Figure 5:**
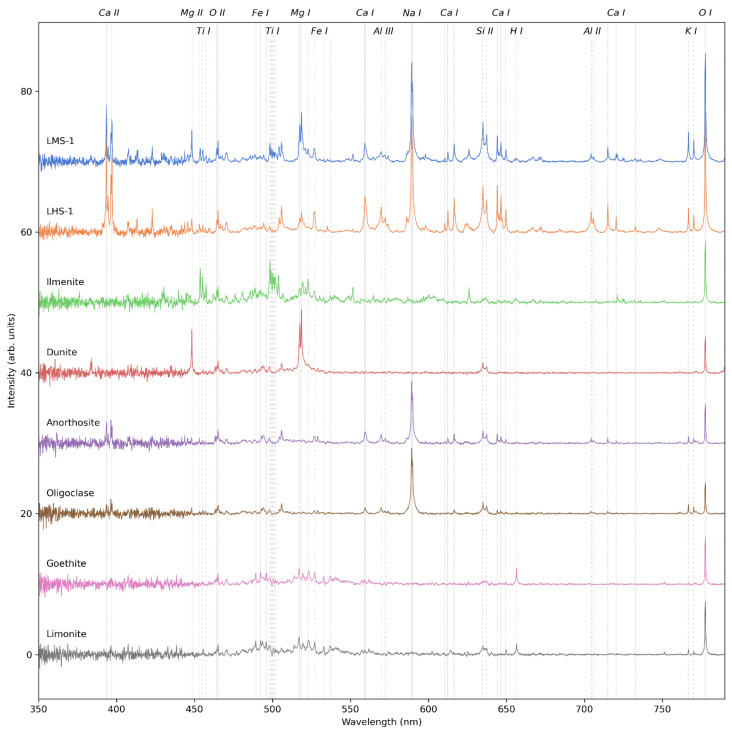
VOILA spectra of Moon analogue samples LMS-1, LHS-1, ilmenite, dunite, anorthosite, oligoclase, goethite, and limonite, plotted with a vertical offset for visual clarity. Each spectrum was obtained by averaging ten individual spectra measured at different positions on the sample. Significant spectral lines are marked by vertical lines.

Figure 5 shows the LIBS spectra of the Moon analogue samples that were investigated in this study (see Table 2) measured with the VOILA breadboard setup. Each spectrum is the average of 10 individual measurements at different positions on the sample. For the two lunar regolith simulants LMS-1 and LHS-1, the individual spectra were acquired by collecting the emissions of 50 successive laser shots in one measurement. For the mineral samples, individual spectra were acquired by collecting the emissions of 30 successive laser shots. All spectra are corrected by the instrument response function and normalized by a factor proportional to the number of laser shots. The strongest emission lines of the plasma species observed in the measured spectra are indicated by vertical dashed lines and are also listed in Table 3.

In the spectra of LMS-1 and LHS-1, spectral lines of all major elements except Fe can be detected with a good signal-to-noise ratio (SNR), while Fe lines are present but comparatively weak. Qualitative differences between the two spectra align with the expected differences in composition: the LMS-1 spectrum has stronger signals of Ti and Mg, while the LHS-1 spectrum has stronger Al, Ca, and Na signals. Since the concentrations of K, Si, and O are similar in both simulants, there is only a small difference in the line intensities of these elements, though these small differences are also in line with the expectations from the regolith compositions.

The spectra of the other geological samples correspond very well with their expected compositions. The ilmenite spectrum consists of intense lines of Fe, Ti, and O. The dunite spectrum does not have a strong Fe signal, but has strong lines of Mg, Si, and O, which is indicative of the Mg-rich Fo93 olivine associated with the sample’s origin in Åheim, Norway. The spectra of anorthosite and oligoclase show the expected lines of Ca, Na, Al, K, Si, and O, while the spectra of goethite and limonite consist of Fe, O, and H lines. These results therefore demonstrate the large potential of LIBS for the identification of lunar geological samples and quantification of elements with state-of-the-art approaches for extraterrestrial in situ LIBS data (e.g., [27]).

It is worth noting that the ratio of ionic to atomic lines in these LIBS spectra is not as high as expected, even though the low pressure at which the measurements were made would favor a higher degree of ionization in the plasma. While some elements such as Si and Al have strong ionic lines, the spectral fingerprint of other elements such as Ca, Ti, Fe, and O is dominated by neutral lines. This indicates that, even if the ambient low-pressure conditions favor a higher ionization, most of the emissions still originate in a cooler plasma region where neutral atoms are prevalent.

### 6.2. Detection of Water Ice in Regolith

**Figure 6:**
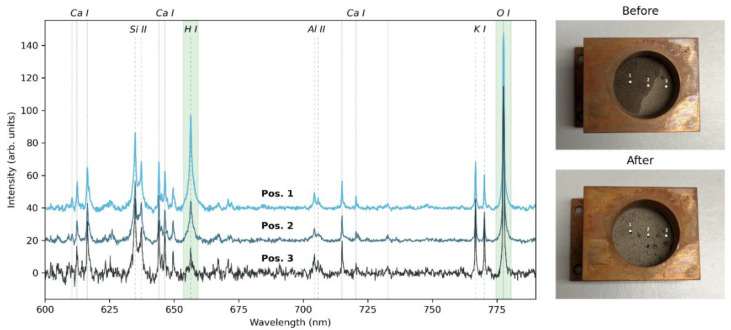
(**left**). VOILA spectra of the lunar regolith mixed with water ice at different positions, where position 1 contains the most water ice and position 3 contains the least. The spectra have been normalized by their total intensity and the spectral lines of H I and O I are highlighted in green. (**Right**): images of the sample before (**top**) and after (**bottom**) freezing and measuring, with labels for the three measurement positions.

For the first investigation into the detection and quantification of water ice, we used a mixture of lunar regolith simulant LMS-1 and water ice. First, LMS-1 was put into a sample holder and lightly pressed to make a smooth surface. Distilled water was then added to one side of the sample holder, so that the water content changes from one side of the sample holder to the other (see Figure 6, top right image). The sample holder was then frozen with liquid nitrogen and placed into the VOILA simulation chamber for the measurements. While the liquid water can be seen clearly, the water ice is not easily visible in the frozen sample (see Figure 6, bottom right image).

On the left side of Figure 6, the LIBS spectra measured with the VOILA breadboard setup in simulated lunar conditions at points 1, 2, and 3 on the sample (as indicated in the images) are shown. The LIBS spectra have been normalized by their total intensity, since the laser ablation is more efficient for points with more water ice due to these areas being more solid than the loose regolith simulant. As expected, the LIBS spectrum of point 1 shows a strong H signal, which is lower for point 2 and lowest for point 3. The O signal varies slightly but remains relatively stable for the different positions, since both LMS-1 and the water ice have a high oxygen content. A summary of the H and O signals and their ratios for each point is given in Table 4, where the signals are the integrals within the highlighted areas in the spectra shown in Figure 6 after subtracting the offset. The H/O signal ratio decreases from position 1 to position 3, indicating that it could be useful as a tracer for water ice presence that is independent of changes in ablation efficiency and overall LIBS intensity.

### 6.3. Detection and Quantification of Water at Low Concentrations

**Figure 7:**
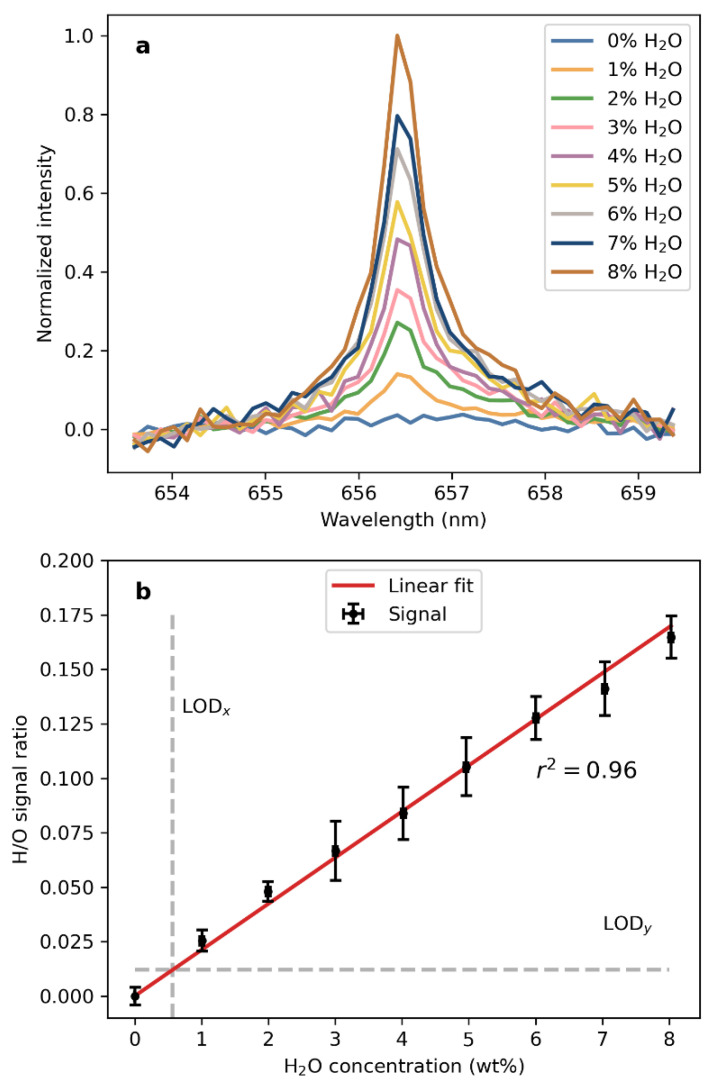
(**a**) H(I) line at 656.46 nm in the LIBS spectra of the samples mixed from CaSO_4_·2H_2_O and Na_2_SO_4_, normalized by the O(I) triplet at 777.5 nm. (**b**) Intensity of the normalized H(I) line independent of the H_2_O concentration in the sample.

The H(I) signals in the mean LIBS spectra of the salt samples used for the investigation of the detection of water at low concentrations are shown in Figure 7a. The spectra have been normalized by the O(I) signal at 777.5 nm, i.e., the spectra were divided by the intensity of the O(I) emission feature that was derived as explained in the previous section. The results show that the signal rises as expected with the increasing water concentration in the samples. At 1% H_2_O in the sample by weight, the H(I) signal already has a SNR of more than 15. The threshold typically used to define the detection of a signal is SNR = 3.3 (e.g., Currie et al., 1968), so that the H(I) signal could be approximately four times lower and still be detected. With this, we can estimate that the VOILA instrument can detect water concentrations of 0.25% by weight, or equivalent hydrogen concentrations of 0.03% by weight.

The H/O signal over the H_2_O concentration in the sample is shown in Figure 7b. The respective line intensities of H(I) and O(I) were obtained in the same way as in the previous section and the signal is the ratio of these line intensities. The error bars indicate the standard deviation obtained from the individual measurements on each sample. The signals follow a clear linear trend, which again shows that the H/O ratio is a suitable tracer for the H_2_O concentration and that a calibration model based on this signal is possible. A correlation coefficient of 0.96 was obtained by simulating a normal distribution with 10 k points and calculating the correlation. Calculating the limit of detection (LOD) as 3*sigma/S, where sigma is the background noise and S is the slope of the linear fit, yields LOD_x = 0.57 wt% H_2_O and is indicated as a vertical line in Figure 7b. The noise was derived from the data of 0% H_2_O.

## 7. Conclusions

VOILA is a prototype LIBS system jointly developed by OHB System AG, Laser Zentrum Hannover e.V., and DLR-OS. It has been optimized for the detection of water and for the geochemical analysis of the lunar surface, in particular the sampling of loose regolith. The results shown here that were obtained with a breadboard model confirm its capability of detecting the major rock-forming elements in lunar analogue samples as well as the capability for water detection and quantification starting from a detection threshold of approximately 0.25% water by weight, or 0.03% hydrogen by weight.

A next step would be the development of prototype hardware moving from a technological readiness level (TRL) 4 towards TRL5. It is likely that the performance can still be increased considerably by improving the optical design and the field of view of the instrument. With hardware closer to the actual flight instrument configuration, more extensive studies on relevant materials should be conducted closely connected to the development of optimized data acquisition procedures and data processing approaches for a calibration of the elements of interest. Additionally, the performance of the hardware in experimentally simulated environmental conditions such as low pressures and low temperatures should be tested eventually to better estimate the instrument’s potential on a lunar robotic mission. Another aspect that was not addressed in this study but should be considered in future works is, moreover, the value or even necessity for calibration with one or several calibration targets.

## Figures and Tables

**Table 1:** Exolith regolith simulant composition as listed on the Exolith website [26].

Oxide	wt% LMS-1	wt% LHS-1
SiO_2_	46.9	51.2
TiO_2_	3.6	0.6
Al_2_O_3_	12.4	26.6
FeO	8.6	2.7
MnO	0.2	0.1
MgO	16.8	1.6
CaO	7	12.8
Na_2_O	1.7	2.9
K_2_O	0.7	0.5
P_2_O_5_	0.2	0.1
LOI *	0.9	0.4
Total **	99	99.4

* Loss on ignition; ** Excluding volatiles and trace elements.

**Table 2:** Geological samples used in the lunar analogue experiments.

Sample	Source	Origin	Major Elements
LMS-1	Exolith	N/A	Si, Ti, Al, Fe, Mg, Ca, Na, K, O
LHS-1	Exolith	N/A	Si, Al, Fe, Mg, Ca, Na, K, O
Ilmenite	Museum für Naturkunde Berlin	Semily, Czechia	Fe, Ti, O
Dunite	Museum für Naturkunde Berlin	Åheim, Norway	Fe, Mg, Si, O
Anorthosite	Museum für Naturkunde Berlin	Ekersund, Norway	Ca, Na, Al, K, Si, O
Oligoclase	Museum für Naturkunde Berlin	Risør, Norway	Ca, Na, Al, K, Si, O
Goethite & limonite	Museum für Naturkunde Berlin	Atterode, Germany	Fe, O, H

**Table 3:** Strongest emission lines of the plasma species observed in the VOILA spectra shown in Figure 5.

Species	Wavelengths (nm)
Si II	634.9, 637.3
Ti I	453.1, 455.1, 457.3, 498.3, 499.2, 500.1, 500.9, 501.6
Al II	704.4, 705.9
Al III	569.8, 572.4
Fe I	489.3, 492.2, 495.9, 522.9, 527.1, 533.0, 537.3
Mg I	517.4, 518.5
Mg II	448.2
Ca I	559.0, 559.6, 610.4, 612.4, 616.4, 644.1, 646.4, 649.6, 715.0, 720.4, 732.8
Ca II	393.5, 397.0
Na I	589.2, 589.8
K I	766.7, 770.1
O I	777.4, 777.6, 777.8
O II	464.0, 464.3, 465.0
H I	656.46

**Table 4:** H and O signals and their ratio in the normalized LIBS spectra from the three positions on the ice/regolith sample.

Position	H Signal (arb. Units)	O Signal (arb. Units)	H/O Ratio
1	87.7	113.0	0.78
2	45.0	97.6	0.46
3	8.0	108.2	0.07

## Data Availability

Data can be made available upon request.
